# Ion Diffusion Reveals Heterogeneous Viscosity in Nanostructured Ionic Liquids

**DOI:** 10.1021/acs.jpclett.4c02996

**Published:** 2024-11-20

**Authors:** Shurui Miao, Amaar Sardharwalla, Susan Perkin

**Affiliations:** Physical and Theoretical Chemistry Laboratory, Department of Chemistry, University of Oxford, Oxford OX1 2JD, U.K.

## Abstract

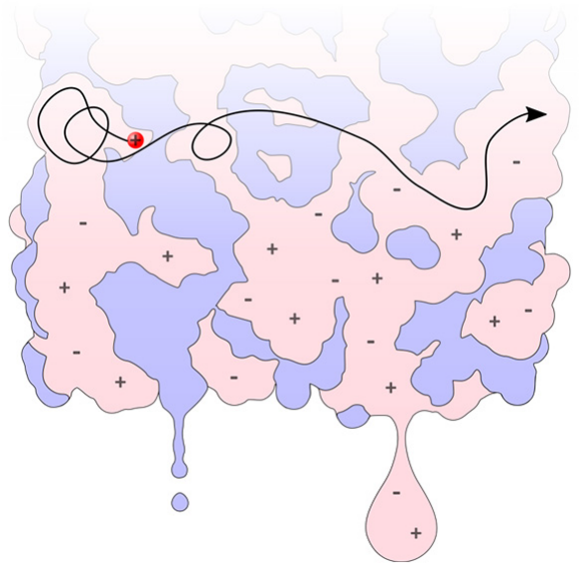

Many ionic liquids (ILs) are composed of interpenetrating polar and apolar networks. These nanoscale networks are sustained by different local intermolecular and electrostatic interactions and are predicted to differ in their physical properties by orders of magnitude. Nonetheless, it is commonplace for the physical properties of ILs to be described by bulk parameters, such as the bulk dynamic viscosity. This study addresses the limitations of using bulk parameter descriptions in nanostructured ILs by applying the Saffman-Delbrück model to interpret the self-diffusion coefficient of ions within the homologous series of [C_n_mim][NTf_2_] ILs. We demonstrate that pulsed field gradient NMR spectroscopy can effectively probe the relative viscosities of polar/charged and apolar networks within these pure ILs. Our calculated polar viscosities show good agreement with literature simulations. Our approach provides valuable insights into the local viscoelastic environments within nanostructured media. This work not only contributes to the understanding of mass and charge transport in ILs but also offers a new experimental perspective for studying structured fluids more broadly.

Ionic liquids (ILs) have gained much interest due to their tunable solvent properties and many potential applications in a wide range of industries.^1−4^ Their versatility is partly due to many combinations of ions that can self-assemble in the bulk to develop an amphiphilic nanostructure.^5^ Driven by a delicate balance of intermolecular and electrostatic interactions, ions can spontaneously rearrange into bicontinuous polar, apolar, and charged networks in the bulk. This allows ILs to solubilize a wide range of solutes and enables new solution processes. While the origin and structural features of the bulk nanostructures have been thoroughly studied,^5^ the diffusion properties of individual ions across such a heterogeneous medium remain poorly understood. This knowledge of charge and mass transport within ILs is critical for its design and application in batteries and as solvents for chemical synthesis and extraction.

Bulk properties such as shear viscosity, density, and self-diffusion coefficients are routinely reported in the literature.^6−8^ However, it is likely an oversimplification to attempt to characterize structurally heterogeneous ILs using a single set of bulk parameters. Studies have confirmed ion mobility in ILs can vary greatly across different length scales and different networks.^9−11^ As an example, the translational relaxation of a given IL exhibits orders of magnitude variability when measured over different length scales.^12^ This highlights the inappropriateness of using a single quantity to describe the multimodal dynamic processes and risks misguiding the design and application of nanostructured ILs. Existing dynamic studies either involve scattering experiments that are challenging to interpret^12,13^ or rely on tracer molecules which potentially disrupt the local nanostructure.^10,14^ Our lack of a reliable experimental approach to determining the physical properties of different networks within a nanostructured IL is becoming one of the major bottlenecks that limit our understanding of how ions and molecules are transported in ILs. This in turn impedes our ability to rationally design ILs with desirable transport properties for many of their long-promised applications.

Computational studies have demonstrated that nanostructured ILs can have two distinct viscosities within the charged/polar networks and the apolar network. The charged/polar network is identified as being stiffer and governs the bulk viscoelastic property of ILs, whereas the apolar network can be orders of magnitude softer.^15,16^ Margulis et al. have quantified this using a tracer molecule that selectively partitioned into either the charged/polar or apolar networks of a nanostructured IL. They reported that tracer mobility was enhanced by diffusion in the soft, apolar domains, while its mobility was reduced in the stiff, polar regions.^10,17−19^ However, the high computational demand requires these studies to forfeit the full electronic details in exchange for sufficient length and time scale for characterizing long-range dynamic processes.^20^ These studies often use tracer species to study transport properties, which potentially induces mechanistic changes by having the tracer act as a buffer species for momentum, volume, and charge conservation.^21,22^ Direct experimental evidence for such heterogeneous transport properties in pure ILs is urgently needed, but it remains elusive due to the complicated nature of these IL nanostructures and how sensitive it is to impurities.^23^

Pulsed-field gradient nuclear magnetic resonance (PG-NMR) is a well-known experimental technique that can reliably measure the self-correlated diffusion coefficients of small molecules in solution.^24^ It is ideal for determining the diffusive behavior of ions in their native environment without the need for introducing tracers. However, literature has extensively reported diffusion-viscosity decoupling in ionic liquids where the measured diffusion coefficient of ions deviates from the prediction by the Stokes–Einstein relationship (eq 1).^14,25−28^ While correlation among ions is known in ILs and causes nonclassical behaviors such as the deviation from the Walden law,^29,30^ the observed diffusion-viscosity decoupling cannot be explained by ion-correlation alone; long-range network and cluster formation are often present.^31−33^ This is a major challenge for interpreting self-correlated diffusion coefficients measured by PG-NMR in the nanostructured media.
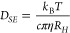
1

Self-diffusion coefficients are commonly interpreted using the Stokes–Einstein equation (eq 1). It relates the self-diffusion coefficient (*D*_*SE*_) to the bulk viscosity of the solution (η) and the hydrodynamic radius (*R*_*H*_) of the diffusing body. The Stokes law assumes an isotropic frictional force is experienced by the diffusing body, but this does not apply to nanostructured ILs with distinctive polar/charged domains and apolar domains.^16^ The translational frictional force that an ion experiences must be different between moving within a domain and moving across domains.^10^ As a result, it is a drastic oversimplification to describe the entire IL medium using a single viscosity parameter such as the Stokes–Einstein equation.

To address this problem, we take inspiration from the field of biophysics, where the anisotropic diffusion of proteins within lipid membranes has been of interest for some time. Saffman and Delbrück (SD) proposed a model (eq 2) to describe the lipid membrane as a thin layer of homogeneous, viscous fluid, with a higher viscosity (η_*m*_) than the surrounding bulk liquid (η_*f*_). This model was successfully applied to evaluate the intramembrane diffusion coefficient of membrane proteins in lipid bilayers:^34,35^
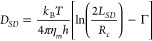
2where *L*_*SD*_ is the Saffman-Delbrück length given by *L*_*SD*_ = *hη*_*m*_/2η_*f*_, η_*m*_ and η_*f*_ are the viscosity of the membrane and surrounding fluid, *h* is the thickness of a lipid monolayer, *R*_*c*_ is the in-plane cross-sectional radius of the diffusing entity, and Γ is the Euler-Mascheroni constant equal to 0.577.^36^ The Saffman-Delbrück model relates the diffusion coefficient of a body embedded in a thin layer of viscous fluid to two distinct viscosities, thereby offering the potential to differentiate the local viscosities of the polar and apolar networks in nanostructured ILs (Figure 1).

**Figure 1:**
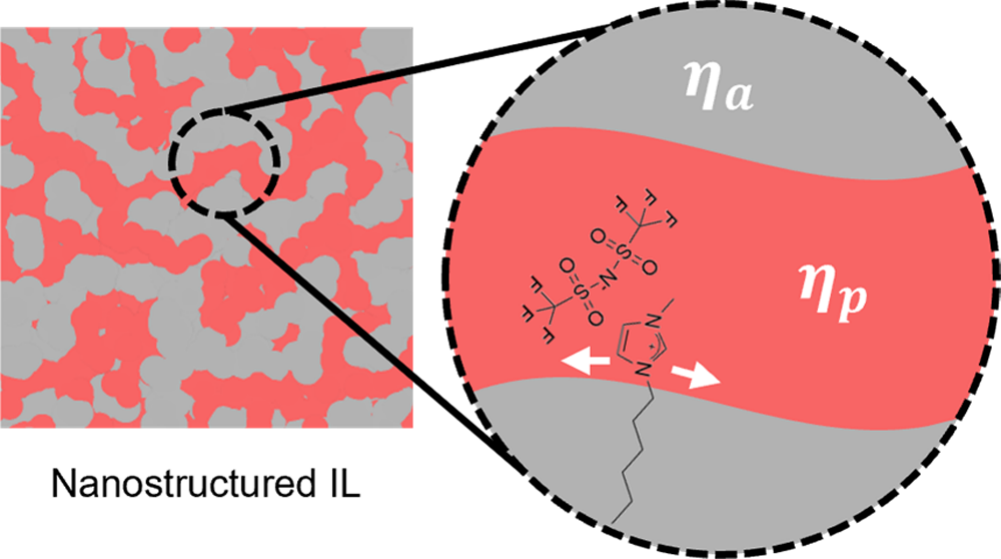
Schematic amphiphilic nanostructure of [C_6_mim][NTf_2_]. The charged/polar network is colored in red, and the apolar network in gray. Locally, the nanostructure is similar to a bilayer structure, and two distinct viscosities are expected to be calculated for each of the two networks.

In order to apply the Saffman-Delbrück model to our nanostructured ionic liquids, we hypothesize that the viscosity of the polar network, η_*p*_, and the viscosity of the apolar network, η_*a*_, replace the membrane and fluid viscosities, respectively. Thus, for ionic liquids, *L*_*SD*_ = *hη*_*p*_/2η_*a*_.

This study aims to assess the applicability of the Saffman-Delbrück model in interpreting the self-correlated diffusion coefficients of ions in nanostructured ILs (Figure 2). These nanostructures are sustained by amphiphilic cations, making them the ideal probe for studying viscosity heterogeneity. The anion self-diffusion is strongly correlated to cation mobility in these ILs.^37^ Therefore, we focused on the more accessible proton NMR, which provides a relatively straightforward way to experimentally determine the relative viscosity of different networks in nanostructured media. We have also performed temperature variation measurements to understand the temperature dependence of the viscosity of the polar networks. This provides an accessible and reliable way to experimentally determine the local viscosity in nanostructured ILs, and it paves the way for understanding how ions and molecules diffuse in nanostructured media in general.

**Figure 2:**
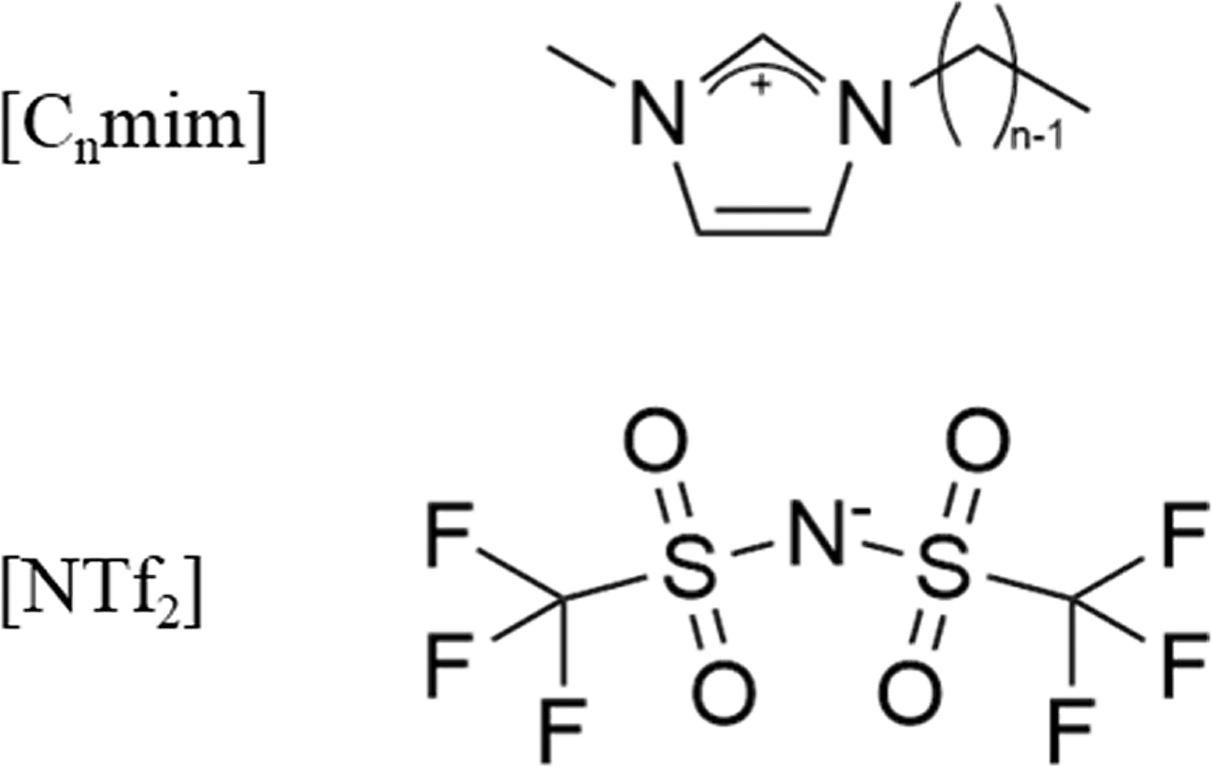
Molecular structure of [C_n_mim][NTf_2_] ILs studied by PG-NMR, where *n* = 2, 3, 4, 6, 7, 10, and 12.

The self-correlated diffusion coefficient of cations in pure ILs is shown in Table 1 and Figure 3 and is consistent with literature values.^38^ We begin by plotting the data in a manner appropriate for comparison to the Stokes–Einstein equation for spherical particles (Figure 3) to highlight how ILs deviate from it. The Stokes–Einstein relationship states that the product of the self-diffusion coefficient, bulk viscosity, and hydrodynamic radius of diffusing particles ((*D*_*NMR*_^*+*^)(η_*Bulk*_)*R*_*H*_) should be constant at a given temperature. However, Figure 3 shows that the [C_n_mim][NTf_2_] ILs exhibit a decreasing trend in this product with increasing alkyl chain length on the cation (n) at 298 K.

**Table 1:** Experimental self-diffusion coefficient of cations (*D*_*NMR*_^*+*^), bulk dynamic viscosity at 298 K (η_*bulk*_),^39^ correlation length of amphiphilic nanostructure (2*h*),^41,50^ bulk viscosity of corresponding *n*-alkanol (η_*n-alkanol*_*= η*_*a*_),^45,46^ and the fitted polar viscosity (η_*p*_) via the Saffman-Delbrück model, of [C_n_mim][NTf_2_] ILs

IL	*D*_*NMR*_^*+*^ (x10^–11^ m^2^s^–1^)	η_*bulk*_ (mPa s)	*2h* (nm)	η_*n-alkanol*_ (mPa s)	η_*p*_ (mPa s)
[C_2_mim][NTf_2_]	4.31	33.0	----	1.1	----
[C_3_mim][NTf_2_]	3.08	45.7	----	2.0	----
[C_4_mim][NTf_2_]	2.32	50.6	1.25	2.5	70.2
[C_6_mim][NTf_2_]	1.43	70.6	1.70	4.6	86.8
[C_7_mim][NTf_2_]	1.14	81.1	1.80	5.8	106.2
[C_10_mim][NTf_2_]	0.757	120.2	2.10	10.9	140.2
[C_12_mim][NTf_2_]	0.475	154.3	2.51	16.6	208.5

**Figure 3:**
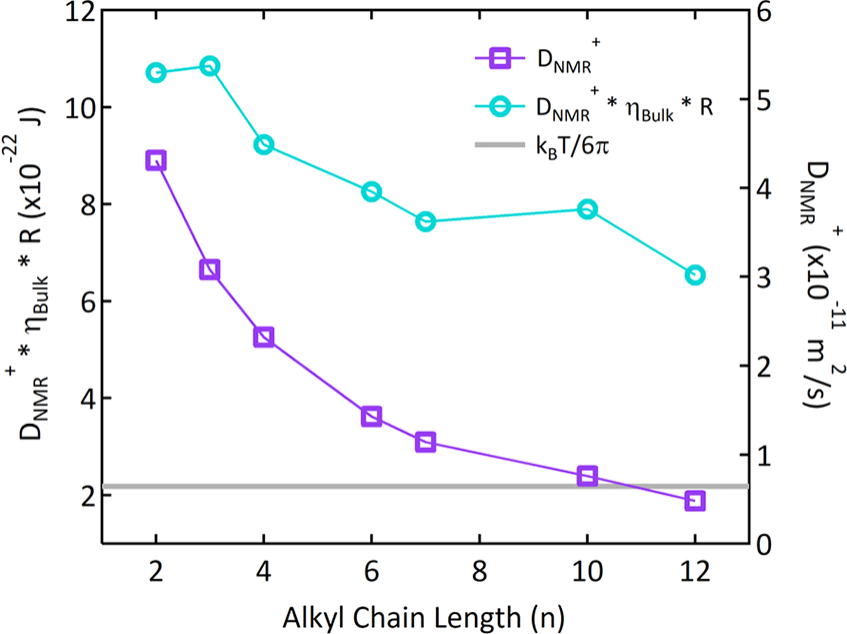
Measured self-correlated diffusion coefficient (*D*_*NMR*_^*+*^) of [C_n_mim]^+^ cations at 298 K (squares, right axis). Errors are smaller than the marker. The product of *D*_*NMR*_^*+*^, bulk viscosity (η_*Bulk*_),^39^ and molecular dimension *R*_*H*_ (circles, left axis), where *R*_*H*_ is calculated from bulk density.^39^ This product, (*D*_*NMR*_^*+*^)(η_*Bulk*_)*R*_*H*_, would be a constant for a fluid obeying the Stokes–Einstein equation (eq 1). Furthermore, for spherical particles, the product (*D*_*NMR*_^*+*^)(η_*Bulk*_)*R* would equal *K*_*B*_*T/6π*, which is shown for 298 K as the gray line.

At short alkyl chain length (n = 2 and 3), the product (*D*_*NMR*_^*+*^)(η_*Bulk*_)*R*_*H*_ is approximately constant. The product (*D*_*NMR*_^*+*^)(η_*Bulk*_)*R*_*H*_ then decreases sharply between n = 3 and 4, after which it decreases more slowly between n = 4 and n = 10. There is another sharper decrease between n = 10 and n = 12. These observations can be connected qualitatively to the known appearance of nanostructure in the bulk fluid of [C_n_mim][NTf_2_] with increasing alkyl chain length from n = 2 to n = 12, as follows. For the smallest cations, n = 2 and n = 3, no nanostructure is present due to the lack of a sufficiently large apolar functional group.^40^ Thus, the product (*D*_*NMR*_^*+*^)(η_*Bulk*_)*R*_*H*_ is relatively constant in this range (although it differs quantitatively from *K*_*B*_*T/6π* due to effects such as the nonspherical and charged nature of the ions). Scattering experiments have demonstrated that, for imidazolium ILs with *n* > 3,^41^ a self-assembled amphiphilic nanostructure forms; sponge-like interpenetrating charged/polar and apolar networks are present up to n = 10.^42^ The onset of nanostructure between n = 3 and n = 4 coincides with a sharp decrease in (*D*_*NMR*_^*+*^)(η_*Bulk*_)*R*_*H*_, which then remains relatively consistent up to n = 10. Beyond n = 10 there is a further structural change, where the interdigitation among long alkyl chains is further enhanced and a bilayer-like nanostructure is observed in the liquid phase.^43,44^ The correlation between changes in the measured (*D*_*NMR*_^*+*^)(η_*Bulk*_)*R*_*H*_ with the known nanostructural evolution of the ILs suggests that ion mobility is less closely tied to bulk properties than to the local environment, which is in turn governed by the amphiphilic nanostructure in ILs.

Next, we adopt the Saffman-Delbrück model (eq 2), previously applied to diffusion within lipid bilayers, and interrogate its ability to account for the viscosity heterogeneity in nanostructured ILs. With increasing alkyl chain length, the effective viscosity of both the polar/charged network and the apolar network is expected to increase due to two considerations. First, the van der Waals interaction among alkyl moieties per alkyl chain increases with chain length. Therefore, the viscosity of the apolar domain will increase as observed in homologous series of *n*-alkanols.^45−47^ The volume change of the apolar domain will reduce the volume fraction of the polar/charged network and impose additional restrictions on the diffusive motion of ions, also increasing the viscosity of the polar/charged network.^48,49^ Second, as discussed earlier, increasing the alkyl group will lead to structural changes in how the ions self-assemble and affect the connectivity of both networks. This will abruptly alter the preferred trajectory for ions to diffuse over large length scales, impacting the measured time-averaged self-diffusion coefficient via PG-NMR.

To apply the Saffman-Delbrück model to ILs, parameters *R*_*c*_ and *h* must be redefined. *R*_*c*_ is defined as the cross-sectional radius of the IL charged group (analogous to the headgroup area of a lipid molecule in earlier use of the SD model) and has a fixed value of 0.275 nm for all [C_n_mim][NTf_2_] ILs, calculated by van der Waals radii of atoms and consistent with experimental measurement.^44^ Analogous to the lipid bilayer, *h* is defined as half of the nanostructure correlation length in the bulk determined by scattering experiments.^41,50^

To account for the varying viscosity of the apolar domains (η_*a*_), the corresponding n-alcohol is used as a reference. Experimental studies have shown the densities of alkyl domains in [C_n_mim][NTf_2_] ILs are similar to their corresponding *n*-alkane.^50^ The addition of a terminal hydroxyl group effectively accounts for the dipolar nature of the [C_n_mim]^+^ cations and enables strong specific interactions between hydroxyl moieties. As a result, large aggregates are also observed in pure *n*-alkanols, similar to ILs,^51,52^ making them ideal reference systems for the apolar domains of [C_n_mim][NTf_2_] ILs. The only unknown in eq 2 is then the polar viscosity, which is predicted to be much higher than the apolar viscosity due to electrostatic interactions.^16^ The fitted value of the polar viscosity is shown in Table 1.

In their computational study of tracer diffusion within 1-butyl-1-methylpyrrolidinium bis(trifluoromethanesulfonyl)imide ([Pyrr_4,1_] [NTf_2_]) at 400 K,^10,18^ Margulis et al. estimated that the diffusion coefficient of a lithium ion is around 0.5 × 10^–10^ m^2^/s. The ion is exclusively confined within the polar/charged network since it is small and has a high charge density. Therefore, the Stokes–Einstein equation can be applied to estimate the polar viscosity of [Pyrr_4,1_][NTf_2_] to be 97.6 mPa s.^10^ The bulk viscosity of [Pyrr_4,1_][NTf_2_] at 400 K is reported to be 4.4 mPa s,^8^ comparable to [C_6_mim][NTf_2_] at the same temperature,^38^ suggesting the two ILs may exhibit similar polar viscosity which is confirmed in our measurement. Similarly, a xenon atom is used as an apolar tracer in the Margulis et al. computational study and yields an apolar viscosity of 1.2 mPa s, also comparable to the bulk viscosity of *n*-alkanol. While a good approximation of the apolar viscosity may not always be possible, the Saffman-Delbrück model can estimate the ratio between the two distinct viscosities present in the nanostructured ILs.

We also investigated the temperature dependence of the polar viscosity for [C_6_mim][NTf_2_] and [C_10_mim][NTf_2_] at 323, 323, and 343 K. The amphiphilic nanostructure in ILs is weakly dependent on temperature.^53,54^ Using the Saffman-Delbrück model, the temperature dependence of the polar and nonpolar viscosities can be distinguished, and thus activation energies for motion in the separate domains are determined; the results are shown in Table 2. The activation energy highlights that the polar viscosity exhibits the strongest temperature dependence in both ILs. More surprisingly, while the viscosity values are different, the activation energy of the polar network is around 45 kJ/mol in both ILs. This suggests the nature of interactions present within the polar network is comparable between the two ILs. This is consistent with their common charge caring groups and highlights the model is sensitive to molecular features that constitute the polar network.

**Table 2:** Experimental self-diffusion coefficient of cations (*D*_*NMR*_^*+*^), bulk dynamic viscosity (η_*bulk*_),^39^ bulk viscosity of 1-hexanol and 1-decanol,^47^ and the fitted polar viscosity (η_*p*_) via the Saffman-Delbrück model of [C_6_mim][NTf_2_] and [C_10_mim][NTf_2_] at 313, 323, and 343 K

		313 K	323 K	343 K
[C_6_mim][NTf_2_]	*D*_*NMR*_^*+*^ (×10^–11^ m^2^ s^–1^)	3.57	5.50	13.0
	η_*bulk*_ (mPa s)	37.5	26.3	14.5
	η_*1-hexanol*_ (mPa s)	2.89	2.21	1.38
	η_*p*_ (mPa s)	34.3	21.4	7.78
	*E*_*a, bulk*_ (kJ mol^–1^)	29.8		
	*E*_*a, 1-hexanol*_ (kJ mol^–1^)	27.1		
	*E*_*a, p*_ (kJ mol^–1^)	45.4		
[C_10_mim][NTf_2_]	*D*_*NMR*_^*+*^ (×10^–11^ m^2^ s^–1^)	2.10	3.70	6.78
	η_*bulk*_ (mPa s)	59.4	39.9	20.6
	η_*1-decanol*_ (mPa s)	6.47	4.69	2.70
	η_*p*_ (mPa s)	40.5	20.4	11.9
	*E*_*a, bulk*_ (kJ mol^–1^)	33.3		
	*E*_*a, 1-decanol*_ (kJ mol^–1^)	28.1		
	*E*_*a, p*_ (kJ mol^–1^)	45.3		

Molecular additives are often used to fine-tune the physical properties of ILs and are known to cause changes in bulk nanostructures. The addition of water and alkanol can disrupt the polar/charged network by competing for hydrogen bonding sites with constituent ions.^55,56^ For example, FTIR and MD studies have shown for χ_*MeOH*_ < 0.8, methanol molecules are primarily solvating IL ions, and no distinct methanol cluster is observed. We investigated the effect of methanol dilution on the self-diffusion of the IL cations. We found that the Saffman-Delbrück model is no longer applicable (see Supporting Information) at χ_*MeOH*_ = 0.4 (<5 wt % methanol), at which no methanol clustering is expected.

The reason for this divergence from the Saffman-Delbrück model when the nanostructure is diluted with methanol can be understood by considering the assumptions underlying the model. The work of Vaz and Dieter showed that the diffusion of lipids in bilayers can only be modeled by the Saffman-Delbrück model if the nonslip boundary condition holds at the surface of the bilayer.^57^ Within the [C_n_mim][NTf_2_] ILs, the nanostructure is sustained by the amphiphilic cation. Its charged headgroup is part of the charged/polar network, and it is covalently linked to its apolar tail, which forms the apolar network (Figure 1). As a result, the nonslip boundary condition is strictly imposed at the interface between the polar and apolar networks within pure ILs. However, with the addition of methanol that solvates IL ions, the nonslip boundary condition is compromised. Some ions can diffuse without being part of the IL nanostructure, generating an effective slip plane. As a result, the measured time-averaged self-diffusion coefficient is significantly faster and the Saffman-Delbrück model cannot account for such excess mobility.

In addition to the nonslip boundary condition, the Saffman-Delbrück model (eq 2) is derived based on two other assumptions. First, the diffusing body is confined to move in a two-dimensional environment for the Euler-Mascheroni constant (Γ) to apply.^34,36^ Computational studies have shown two distinct models of motion exist for tracers that selectively partition into one of the networks.^10,18^ The tracer mostly undergoes Brownian motion within the preferred network and occasionally jumps to nearby domains of similar polarity. This violates the requirement for the translational motion to be confined within a two-dimensional plane. Therefore, the Saffman-Delbrück model should not be applied to interpret the translational self-diffusion coefficient of tracer species. Nevertheless, when the diffusion of an amphiphilic ion is measured, it must be situated at the boundary of the polarity domains and will likely undergo two-dimensional motions, supporting the use of this model for understanding pure ILs.

Second, the Saffman-Delbrück length is assumed to be significantly larger than the cross-sectional radius of the diffusing body (i.e., *L*_*SD*_*≫ R*_*c*_).^58^ For the well-established application of the model, the *L*_*SD*_*/R* ratio is typically in the range of thousands due to the large viscosity difference between lipid membrane and aqueous solutions.^58^ However, in nanostructured ILs, the viscosity difference between the polar/charged network and the apolar network is only 1 order of magnitude.^10^ This implies that the extended Saffman-Delbrück model proposed by Petrov and Schwille is potentially more suitable for describing ILs.^58^ Nevertheless, given many other key factors such as the precise dimension of IL nanostructures (i.e., *h*) and the local curvature are either estimated or unaccounted for,^17,59^ the classic Saffman-Delbrück equation is sufficient to allow a semiquantitative interpretation of self-diffusion coefficients in nanostructured ILs.

In nanostructured ionic liquids, where distinct polar/charged and apolar networks are present, it is an oversimplification to describe the medium using one set of bulk parameters. This is reflected in the breakdown of the Stokes–Einstein equation, often reported in attempts to understand charge and mass transport properties and mechanisms in these nanostructured ILs. Meanwhile, many computational and experimental studies have highlighted the viscosity heterogeneity in such media, where the intertwined networks can be orders of magnitude different in local viscosity. Therefore, it is crucial to be able to measure and compare the different viscosities, which will undoubtedly affect how ions and neutral molecules diffuse within ILs. We have applied the classic Saffman-Delbrück model, originally derived to describe Brownian motion within lipid membranes, to a homologous series of [C_n_mim][NTf_2_] ILs with varying nanostructure. We showed that the self-diffusion coefficient obtained by standard pulsed field gradient NMR spectroscopy can be used to probe the relative viscosity of the polar/charged network and the apolar network. Our calculated polar viscosities agree with the estimated values by simulations. Furthermore, we performed variable-temperature and dilution experiments to test the robustness of the Saffman-Delbrück model. We discovered that it can be applied at various temperatures, as long as the underlying liquid nanostructure is not disturbed. On the other hand, it was found that dilution of the IL with methanol rendered the model inapplicable. We suspect this is due to the incorporation of methanol into the polar network, greatly reducing its viscosity and rendering the Saffman-Delbrück length closer to the molecular dimension, thereby invalidating the assumptions underlying the model. To address this, the extended Saffman-Delbrück model is of use. While the present study is limited to being semiquantitative, we have demonstrated that by adopting a new mathematical model, PG-NMR can be used as a reliable and accessible technique to provide information regarding the local environment of ions and solutes within nanostructured media. The insights obtained may have impacts beyond the ionic liquids studied, toward the study of the transport properties of molecules and ions within structured fluids more generally.

## Supporting Information Available

Experimental methods, methanol dilution, raw data (PDF)
