# Supplementary material for: Ion Diffusion Reveals Heterogeneous Viscosity in Nanostructured Ionic Liquids
Source: J Phys Chem Lett. 2024 Nov 20;15(47):11855–61. doi: 10.1021/acs.jpclett.4c02996 (PMC11613658; doi:10.1021/acs.jpclett.4c02996)
Supplement: Supplementary file 1 — jz4c02996_si_001.pdf [file jz4c02996_si_001.pdf]

Ion Diffusion Reveals Heterogeneous Viscosity in Nanostructured Ionic Liquids

Shurui Miao[*a], Amaar Sardharwalla[a], Susan Perkin[a]*

[a] Physical and Theoretical Chemistry Laboratory, Department of Chemistry, University of Oxford, OX1 2JD, UK

shurui.miao@chem.ox.ac.uk

Materials

All [$C_n$mim][NTf_2] ILs (molecular structure shown in Figure 2) were purchased from IoLiTech GmbH with the highest purity (> 99 %). ILs were dried overnight on a Schlenk line at 40 ˚C and 10^{-5} atm. Prior to NMR, water contents were determined using Karl-Fischer Titration (Hanna Instruments, HI934) to be < 500 ppm, and no impurity was observed in any of the ^{1}H NMR spectra. HPLC-grade methanol was purchased (Merck, > 99.9 %) and used as a molecular diluent.

PG-NMR

Pulsed-field gradient Nuclear Magnetic Resonance Spectroscopy (PG-NMR) was performed using Bruker NEO 400 MHz or AVIII HD 500 MHz spectrometers with 5 mm z-gradient broadband multinuclear SMART probe and 5 mm triple-resonance TBO probe respectively. The built-in method of Diffusion Ordered Spectroscopy (DOSY) with bipolar gradients and

longitudinal eddy current delay (ledbpgp2s) pulse sequence was used to extract the diffusion coefficient of each ^1H environment on the cation. All dried samples were injected in standard 5 mm diameter tubes under nitrogen and sealed with Teflon tapes to prevent moisture uptake. The diffusion measurements were performed after careful calibration of the pulses, determination of the longitudinal relaxation time, and maximised signal strength. A pair of diffusion time (Δ) and duration of the pulsed field gradient (δ) were found for each sample to ensure sufficient signal attenuation (< 30%) for all proton environments. The pulsed field gradient varied from 10 to 90 % of the probe's maximum gradient strength (50.1 G/cm) in quadratic spacing. 16 spectra were recorded at different strengths of the pulsed field gradient, each consisting of 16 experiments. The relaxation delay between each experiment was 5 times the measured longitudinal relaxation time. Only the ^1H nucleus was used to determine the cation self-diffusion coefficient since it is the cation in [$C_n$mim][NTf_2] ILs that sustain the nanostructure. Self-diffusion of anions can be measured using ^{19}F NMR, however, studies have also shown that anion and cation diffusion coefficients are strongly correlated (e.g. Ref. 37). Therefore, we expect the anion to follow the same trend as the cation and will not contribute new insight to the fitting.

The 500 MHz spectrometer is also equipped with a BCU-II temperature regulation unit for making variable temperature measurements between 298 and 343 K (fluctuation < 0.1 K). To minimise the effect of Rayleigh-Bénard (vertical) and Hadley (horizontal) convection due to temperature gradients, samples were equilibrated for at least 30 min inside the spectrometer before measurements. A more complex convection-compensating pulse sequence was used for all measurements above 298 K.[1]

Measured signals (peak height) are attenuated by the translational motion of molecules, and it is quantitatively described by the Stejskal-Tanner equation (Eq. S1):

$$I_G = I_0 e^{\left[-(\gamma\delta G)^2 D\left(\Delta-\frac{\delta}{3}\right)\right]} \qquad \text{Eq.S1}$$

Where I_G is the signal intensity at gradient strength (G), I_0 is the signal intensity at a gradient strength of zero, γ is the gyromagnetic ratio of the observed nucleus, D is the diffusion coefficient, δ is the gradient pulse duration, and Δ is the diffusion time. During the DOSY experiment, γ, δ, and Δ are optimised and fixed, and gradient strength is the only variable. Linear fit on a log-linear plot was performed to extract the self-diffusion coefficient of the cation (D_{NMR^+}). δ and Δ are manually optimised for each ionic liquid (at each temperature) to achieve optimal signal-to-noise ratio and the best G-dependent signal attenuation for all proton environments. Multiple sets of δ and Δ were used to extract the self-diffusion coefficient of the cation in [C_4mim][NTF_2], values obtained are consistent and match literature values.

Addition of Methanol

Electrochemical and NMR experiments discovered that the added water can screen electrostatic interactions and significantly enhance the translational relaxation of the charged tracer and IL itself.[2, 3] To further assess the applicability of the Saffman-Delbrück model, methanol was added to [C_6mim][NTf_2] and [C_{10}mim][NTf_2] at 0.4, 0.6, 0.8, and 0.95 mole fraction (χ_{MeOH}). Methanol concentrations are chosen to represent different IL-solvent structures. FTIR and MD study has shown for $\chi_{MeOH} < 0.8$, methanol molecules are primarily solvating IL ions, and no distinct methanol cluster is observed. With further addition of methanol to $\chi_{MeOH} > 0.9$, the IL network is rendered to ion pairs and the mixture adopts methanol-like properties.[4] The measured self-diffusion coefficient of the cations is shown in Table S1, viscosity was measured using Anton Paar rolling-ball viscometer (Lovis 2000

M/ME). The Saffman-Delbrück model (Eq. 2) is applied to estimate the polar viscosity upon methanol dilution. Results are shown in Figure S1, where no real solution can be found. A numeric solution is only attainable when a smaller constant is used in replacement of the Euler-Mascheroni constant (Γ).

Table S1. Experimental composition, bulk dynamic viscosity at 298 K (η_{bulk}), correlation length of amphiphilic nanostructure in pure ILs ($2h$),[5, 6] bulk viscosity of corresponding n-alkanol ($\eta_{n\text{-}alkanol} = \eta_a$),[7] and the self-diffusion coefficient of cations (D_{NMR}^+) measured via PG-NMR.

IL	χ_{MeOH}	*Methanol (wt %)*	η_{bulk} (mPa s)	$2h$ (nm)	$\eta_{n\text{-}alkanol}$ (mPa s)	D_{NMR}^+ ($\times 10^{-11}$ m^2s^{-1})
$[C_6mim][NTf_2]$	0.4	4.6	22.5	1.70	4.6	5.61
	0.6	9.7	10.9			9.28
	0.8	22.3	3.9			22.9
	0.95	57.6	1.1[a]			70.1
$[C_{10}mim][NTf_2]$	0.4	4.1	36.1	2.10	10.9	3.76
	0.6	8.7	17.4			5.65
	0.8	20.3	6.0			13.0
	0.95	54.7	1.3[a]			42.0

[a] Values are near the lower limit of the instrument and has large uncertainties.

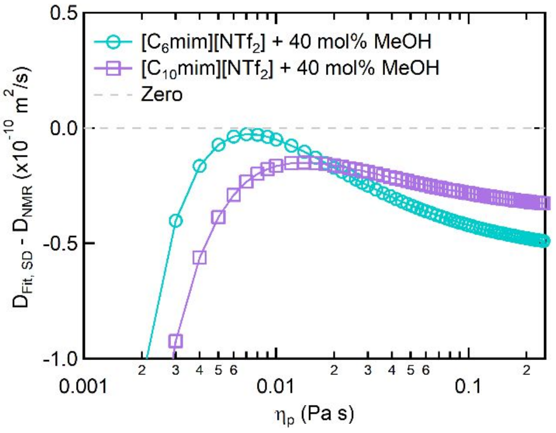

Figure S1. Attempt to solve the Saffman-Delbrück equation for $[C_6mim][NTf_2]$ and $[C_{10}mim][NTf_2]$ + 0.4 mole fraction of methanol using experimental data shown in Table S1. A numerical solution will be the x-intercept. No real solution can be found using the Euler-Mascheroni constant ($\Gamma = 0.577$) for both mixtures.

REFERENCES

(1) Claridge, T. D. W. Chapter 10 - Diffusion NMR Spectroscopy. In *High-Resolution NMR Techniques in Organic Chemistry (Third Edition)*, Claridge, T. D. W. Ed.; Elsevier, 2016; pp 381-419.

(2) Schröder, U.; Wadhawan, J. D.; Compton, R. G.; Marken, F.; Suarez, P. A. Z.; Consorti, C. S.; de Souza, R. F.; Dupont, J. Water-induced accelerated ion diffusion: voltammetric studies in 1-methyl-3-[2,6-(S)-dimethylocten-2-yl]imidazolium tetrafluoroborate, 1-butyl-3-methylimidazolium tetrafluoroborate and hexafluorophosphate ionic liquids. *New Journal of Chemistry* **2000**, *24*, 1009.

(3) Cascão, J.; Silva, W.; Ferreira, A. S. D.; Cabrita, E. J. Ion pair and solvation dynamics of [Bmim][BF4] + water system. *Magnetic Resonance in Chemistry* **2018**, *56* (2). DOI: 10.1002/mrc.4673.

(4) Roth, C.; Appelhagen, A.; Jobst, N.; Ludwig, R. Microheterogeneities in Ionic-Liquid–Methanol Solutions Studied by FTIR Spectroscopy, DFT Calculations and Molecular Dynamics Simulations. *ChemPhysChem* **2012**, *13* (7). DOI: 10.1002/cphc.201101022.

(5) Triolo, A.; Russina, O.; Bleif, H. J.; DiCola, E. Nanoscale Segregation in Room Temperature Ionic Liquids. *The Journal of Physical Chemistry B* **2007**, *111* (18), 4641-4644. DOI: 10.1021/jp067705t.

(6) Pontoni, D.; Haddad, J.; Michiel, M. D.; Deutsch, M. Self-segregated nanostructure in room temperature ionic liquids. *Soft Matter* **2017**, *13* (38), 6947-6955. DOI: 10.1039/C7SM01464C.

(7) Haynes, W. M. *CRC Handbook of Chemistry and Physics*; CRC Press, 2017.
